# Intent to Adopt Video-Based Integrated Mental Health Care and the Characteristics of its Supporters: Mixed Methods Study Among General Practitioners Applying Diffusion of Innovations Theory

**DOI:** 10.2196/23660

**Published:** 2020-10-15

**Authors:** Markus W Haun, Isabella Stephan, Michel Wensing, Mechthild Hartmann, Mariell Hoffmann, Hans-Christoph Friederich

**Affiliations:** 1 Department of General Medicine and Psychosomatics Heidelberg University Heidelberg Germany; 2 Department of General Practice and Health Services Research Heidelberg University Heidelberg Germany

**Keywords:** video consultations, videoconferencing, telehealth, integrated care, mental health, preimplementation, diffusion of innovations, early adopters, mixed methods, cumulative logit model, content analysis

## Abstract

**Background:**

Most people with common mental disorders, including those with severe mental illness, are treated in general practice. Video-based integrated care models featuring mental health specialist video consultations (MHSVC) facilitate the involvement of specialist mental health care. However, the potential uptake by general practitioners (GPs) is unclear.

**Objective:**

This mixed method preimplementation study aims to assess GPs’ intent to adopt MHSVC in their practice, identify predictors for early intent to adopt (quantitative strand), and characterize GPs with early intent to adopt based on the Diffusion of Innovations Theory (DOI) theory (qualitative strand).

**Methods:**

Applying a convergent parallel design, we conducted a survey of 177 GPs and followed it up with focus groups and individual interviews for a sample of 5 early adopters and 1 nonadopter. We identified predictors for intent to adopt through a cumulative logit model for ordinal multicategory responses for data with a proportional odds structure. A total of 2 coders independently analyzed the qualitative data, deriving common characteristics across the 5 early adopters. We interpreted the qualitative findings accounting for the generalized adopter categories of DOI.

**Results:**

This study found that about one in two GPs (87/176, 49.4%) assumed that patients would benefit from an MHSVC service model, about one in three GPs (62/176, 35.2%) intended to adopt such a model, the availability of a designated room was the only significant predictor of intent to adopt in GPs (β=2.03, SE 0.345, *P*<.001), supporting GPs expected to save time and took a solution-focused perspective on the practical implementation of MHSVC, and characteristics of supporting and nonsupporting GPs in the context of MHSVC corresponded well with the generalized adopter categories conceptualized in the DOI.

**Conclusions:**

A significant proportion of GPs may function as early adopters and key stakeholders to facilitate the spread of MHSVC. Indeed, our findings correspond well with increasing utilization rates of telehealth in primary care and specialist health care services (eg, mental health facilities and community-based, federally qualified health centers in the United States). Future work should focus on specific measures to foster the intention to adopt among hesitant GPs.

## Introduction

### Telehealth in General Practice Mental Health

Most people with common mental disorders and many of those with severe and enduring mental illness are treated within general practice [1-3]. For example, according to German health insurance claims data, one in every 2 patients with two or more mental health conditions is treated by general practitioner (GP) only [4]. By increasing the access to specialist care, integrated care models are effective in ensuring seamless care trajectories [5-8]. However, in many remote and rural areas, mental health specialists (MHS), who play a pivotal role in these models, are not readily available [9]. Moreover, patients, particularly those with long-term conditions, struggle with long travel distances [10-12]. Hence, video-based integrated care models have been introduced to overcome the limitations of face-to-face models and have proven to be safe and equally effective [13-18]. Although telemedicine in mental health is relatively common compared with other specialties, only 12.7% of all GPs use video consultations in their practice [19,20]. GPs are concerned with increased workload and the lack of reimbursement and training [21,22].

### GPs as Early Adopters of Telehealth

In the Diffusion of Innovations (DOI) theory, innovativeness is defined as the degree to which an individual is relatively early in adopting new practices compared with other members of a social system [23,24]. To this end, GPs can be characterized by the extent to which they are open to the implementation of new technologies [25,26]. Specifically, GPs can be placed on a spectrum running from early adopters to the so-called laggards (nonadopters) [27]. Putting aside the uncertainty, early adopters show a more favorable attitude toward change and science, less dogmatism, and a greater knowledge of innovations. Currently, little is known about the innovativeness of GPs with respect to telehealth applications such as video consultations. Specifically, the characteristics of GPs with early intent to adopt are unknown. In this regard, we conducted a systematic search in MEDLINE (Medical Literature Analysis and Retrieval System Online) and Web of Science from inception to August 19, 2020 (Multimedia Appendix 1 [28]). Among the 3944 records, we found 5 records concerning the early adoption of telehealth interventions in general practice. We identified 1 conceptual article [29], 1 study protocol [30], 1 case study of an early adopter site [31], and 2 qualitative studies [32,33]. The case study describes the impact of a web-based consultation system on working practices in an inner-city general practice. Overall, evidence on anticipated efficiency gains was scarce [29]. One qualitative interview study investigated the perceptions of 9 Norwegian GPs toward the use of 4 digital health services for patients (electronic booking to schedule visits, electronic prescriptions, text-based nonclinical inquiries, and text-based electronic consultation). Besides skepticism about the clinical utility of e-consultations, GPs entertained concerns that elderly patients, people unfamiliar with technology, and some patients receiving psychiatric care required traditional face-to-face alternatives. None of the studies determined the proportion of GPs intending to adopt mental health specialist video consultations (MHSVC). Predictors of early adoption and characteristics of GPs with early intent to adopt also remain unclear. However, evidence on both the early adopter proportion and predictors, along with the characteristics of GPs with early intent to adopt, is needed to efficiently promote and implement telehealth applications in general practice [34].

### Rationale of the Study

This mixed method study aims to (1) assess GPs’ intent to adopt MHSVC in their practice, (2) identify predictors for early intent to adopt (quantitative strand), and (3) characterize GPs with early intent to adopt based on the DOI (qualitative strand) theory by Everett Rogers. Specifically, we conducted a survey followed by focus groups and interviews with GPs as part of the preimplementation phase of the PROVIDE (ImPROving cross-sectoral collaboration between primary and psychosocial care: An implementation study on VIDEo consultations) project [35,36]. PROVIDE features a service model in which GPs refer patients with depression and/or anxiety to video consultations conducted in their practice with a remotely located MHS. The model comprises up to 5 MHSVC sessions that focus on specialized clinical evaluation (systematic assessment and diagnostics), brief therapy (general support, brief psychotherapy, and psychopharmacology), and, if required, triage to specialist mental health service.

## Methods

### Mixed Methods Study Design

We applied a convergent parallel design to gain an understanding of GPs’ attitudes toward adopting MHSVC. This design allows for the collection and analysis quantitative and qualitative data followed by an integration of both. Specifically, we started with the collection and analysis of cross-sectional survey data and followed it up with the collection and analysis of the qualitative focus group and interview data [37]. The quantitative strand comprised (1) the estimation of the anticipated benefit, acceptability, and intent to adopt MHSVC among GPs, (2) the exploration of predictors for the intent to adopt, and (3) the identification of supporters in this population of interest. The subsequent qualitative strand included the in-depth characterization of these supporters, whose sampling was informed by the quantitative results. Specifically, the composition of the qualitative sample resulted from a direct interaction between the two strands (point of interface) [38].

This study received ethical approval from the Ethics Committee of the Medical Faculty at the University of Heidelberg (Reference: S-197/2017) and was preregistered with the German Clinical Trials Register (DRKS00012487). We applied the COnsolidated criteria for reporting qualitative research (COREQ; Multimedia Appendix 2).

### Setting

The PROVIDE research group at Heidelberg University in Heidelberg, Germany coordinated and conducted the survey, the focus groups and the telephone interviews. Recruitment and data collection lasted from May 2017 to June 2017 for the survey and from July 2017 to August 2017 for the focus groups and interviews, respectively.

### Participants

We invited all GPs registered with the Association of Statutory Health Insurance Physicians in 1 urban and 4 rural districts (from a total of 35 districts in Baden-Wuerttemberg, one of 16 German federal states) to participate in the study. Apart from registration, there were no other eligibility criteria for GPs. All the GPs received a personalized cover letter, a 4-page leaflet containing information about the study, including the MHSVC care model, and a questionnaire on the intent to adopt (Multimedia Appendix 3), which the GPs were asked to send back by fax. We reminded all nonresponders with up to 3 follow-up phone calls. We did not offer any incentive for answer the questionnaire. A total of 41 GPs declared interest in participating in the focus groups. We conducted 4 focus groups (range: 2-6 participants, 90-120 min) involving 16 GPs at Heidelberg University Hospital. One GP, who had been invited by another GP, participated without a formal invitation. Whenever possible, we opted for focus groups that facilitated less constrained discussions for capturing a broad range of perceptions [39,40]. We conducted individual telephone interviews with 3 GPs (40-55 min) who were eventually unable to attend focus groups. We offered a nonadvertised individual monetary compensation of €50 (US $58) to each participant. A total of 16 GPs refused to participate, mostly because of holiday leave (n=4) and lack of interest (n=4). A total of 7 GPs were not contacted because of the earlier-than-expected data saturation. The initial analysis based on the data of all 19 participants focused on the overall potential for integrating MHSVC in general practice. The findings were published elsewhere [35]. For this study, we limited our analysis to 6 focus group and interview participants who had (1) also participated in the initial survey and (2) were identified as supporters and nonsupporters.

### Data Sources and Measurement

We developed a brief 12-item self-completion, written questionnaire (Multimedia Appendix 3). It contained 3 domains: (1) demographic data of the GP, (2) characteristics of the practice, and (3) intent to adopt MHSVC (anticipated benefit for patients, acceptance of MHSVC, and intent to adopt). We only used closed questions with precoded response options. To ensure content validity, we piloted the questionnaire to an experienced GP and a senior health services researcher to check for the unambiguous meaning of instructions and questions, along with sufficiency of the response categories available. The degree of urbanization of the area practices was stratified according to the current standard established by the European Commission [41].

To prompt group discussions and interviews, we developed a semistructured question guide (Multimedia Appendix 4). The questions focused on how GPs perceived current health care for patients with mental disorders, the potential for integrating MHSVC into office-based routine general practice, and the determinants of the implementation of MHSVC. We piloted the guide to one GP and one senior health services researcher, and it was also reviewed after the first focus group. After obtaining written informed consent from all participants, M Haun (internal medicine specialist, senior researcher, and content expert for mental health services) and M Hoffmann (sociologist, PhD student, and expert in qualitative research) moderated the focus groups. To stimulate the discussion, the moderators presented a 7-min video clip illustrating the MHSVC model. We also compiled field notes during all focus groups and interviews. Qualitative data were audio-recorded and uploaded to a secure server of Heidelberg University Hospital, which was accessible only to the research team. We stopped data collection when no new insights emerged from the data, suggesting that we had achieved saturation of content and a rich description through a variety of codes and associated meanings [42].

### Statistical Analysis (Quantitative Strand)

**Figure 1:**
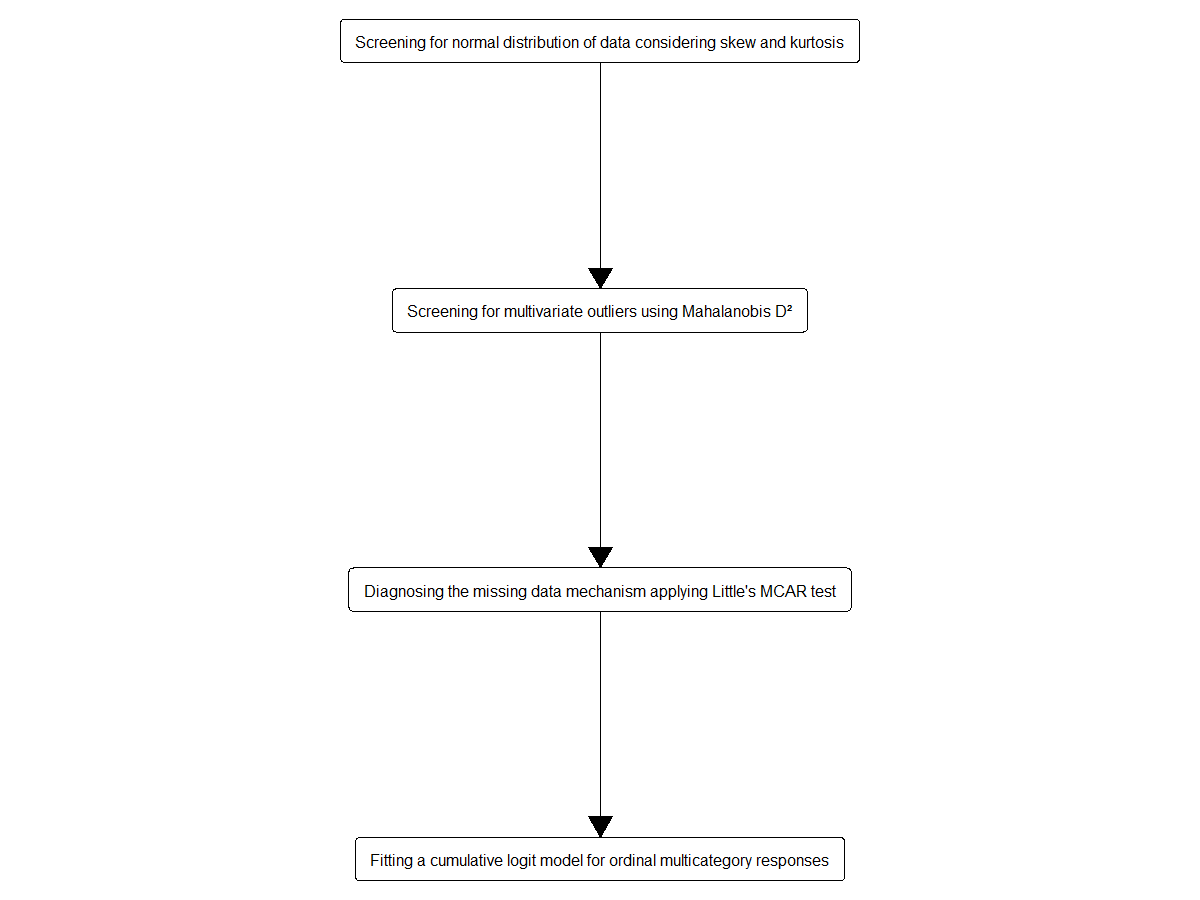
Overview of the statistical analysis. MCAR: missing completely at random.

An overview of the statistical analysis is provided in Figure 1. The data preparation for the multivariate analysis included screening for normality and outliers. First, we inspected univariate distributions assuming multivariate normality if skewness and kurtosis item and score values fell within the normal range (2 to 2 and 7 to 7, respectively). In addition, we computed Mahalanobis D²; outliers were deleted before subsequent analyses. Second, Little’s test of missing completely at random (R package *BaylorEdPsych*) indicated a missing-completely-at-random pattern (*χ^2^*_2_=1.3; N=176; *P*=.53) and a maximum fraction of missing information of 2.8% (5/177) at the item level. Hence, we refrained from imputing missing data, assuming comparable efficiency for *the available case analysis. Finally, we fitted a cumulative logit model for ordinal multicategory responses with proportional odds structure (R package VGAM*) to the data using the intent-to-adopt item 11 as the dependent variable and 6 GP and practice-related predictors (age of the GP, additional mental health care qualification of the GP, degree of urbanization of the area that the practice was in, practice type (single or group or shared), average number of treated cases, and availability of a room designated for video consultations). We tested the proportional odds assumption, that is, the effect of an independent variable would be uniform for all levels of the intent-to-adopt item 11 as the dependent variable, using the likelihood ratio test from the ordinal package [43]. Concerning the qualitative analysis, we considered all GPs to be supporters who fully agreed on the intent-to-adopt item 11 (*In principle, can you personally imagine providing video consultations conducted by MHS to patients with mental disorders in your practice?*). Similarly, we identified all GPs as nonsupporters (1) who fully disagreed on item 11 and (2) rather or fully disagreed on item 10 (*Would you support the idea of treating patients with mental health disorders through video consultations conducted by MHS in primary care practices?*). The statistical analysis was conducted independently by 2 analysts (M Haun and Justus Tönnies, MSc) using R, version 4.0.2 [44]. For all analyses, statistical significance was evaluated at a type 1 error of 5% (two-tailed).

### Content Analysis (Qualitative Strand)

Before anonymizing the data, a professional transcription service conducted verbatim audio transcriptions of the recordings. The aim of the qualitative analysis was to find common characteristics across the 5 supporters and to interpret these findings using DOI [24]. Thus, we accounted for differences and similarities between the supporters and nonsupporters of the MHSVC and the generalized categories of early adopters and nonadopters (so-called laggards), as proposed in DOI. Therefore, we conducted an inductive content analysis with inductive or bottom-up development of the coding system in MAXQDA, version 18 [45]. For data collection, analysis, and interpretation, we followed the principle of investigator triangulation (Table 1) to limit potentially prevailing researcher biases by leveraging multidisciplinary expertise [46]. First, to gain an initial understanding of the data, two coders (IS and M Hoffmann) independently read one transcript, highlighting the most important passages. Second, to facilitate the comparison of the major topics, each researcher defined codes that represented the highlighted key aspects (IS and M Hoffmann). Third, both coders compared their analyses, discussed disagreements, and resolved them (IS and M Hoffmann). Fourth, both researchers independently applied the new coding system to another transcript and reviewed their findings (IS and M Hoffmann). To ensure that all key aspects were represented in the coding system, codes were continuously modified when new aspects emerged. Finally, IS analyzed the remaining transcripts and met with M Hoffmann and M Haun to check the coding system for inter-coder consistency and discuss its validity (Multimedia Appendix 5; IS, M Hoffmann, and M Haun). All researchers involved in the investigator triangulation checked the final interpretation of the data for completeness and cohesiveness.

**Table 1:** Details for investigator triangulation.

Characteristics	M Hoffmann	IS	M Haun
Disciplinary background	Sociologist	Medical student, final year elective	MD, psychologist, internal medicine specialist, attending physician in psychosomatic medicine
Training and expertise	Early career researcher, >6 years of experience with qualitative methods	Early career researcher	Senior researcher, >10 years of experience with quantitative and qualitative methods
Epistemological stance	Critical realist	Critical realist	Critical realist
Role	PhD student in the PROVIDE^a^ project	MD student in the PROVIDE project	Principal investigator of the PROVIDE project
Stages involved or points of collaboration (Degree of investigator independence)	Collection of quantitative and qualitative data (moderate), cleaning of qualitative data (high), inductive content analysis (high), development of a joint coding system (moderate to high)	Cleaning of qualitative data (high), inductive content analysis (moderate), development of joint coding system (moderate)	Collection of quantitative and qualitative data, quantitative data analysis (high), content analysis: review of initial coding systems (high), content analysis: arbiter for developing a joint coding system (moderate to high)
Statement of investigator triangulation impact	Overall, investigator triangulation contributed to (1) consensus reaching on divergent views or interpretations and (2) confirmation of codes and themes which covered the data quite completely and cohesively.	Overall, investigator triangulation contributed to (1) consensus reaching on divergent views or interpretations and (2) confirmation of codes and themes which covered the data quite completely and cohesively.	Overall, investigator triangulation contributed to (1) consensus reaching on divergent views or interpretations and (2) confirmation of codes and themes which covered the data quite completely and cohesively.

^a^PROVIDE: ImPROving cross-sectoral collaboration between primary and psychosocial care: An implementation study on VIDEo consultations.

## Results

**Figure 2:**
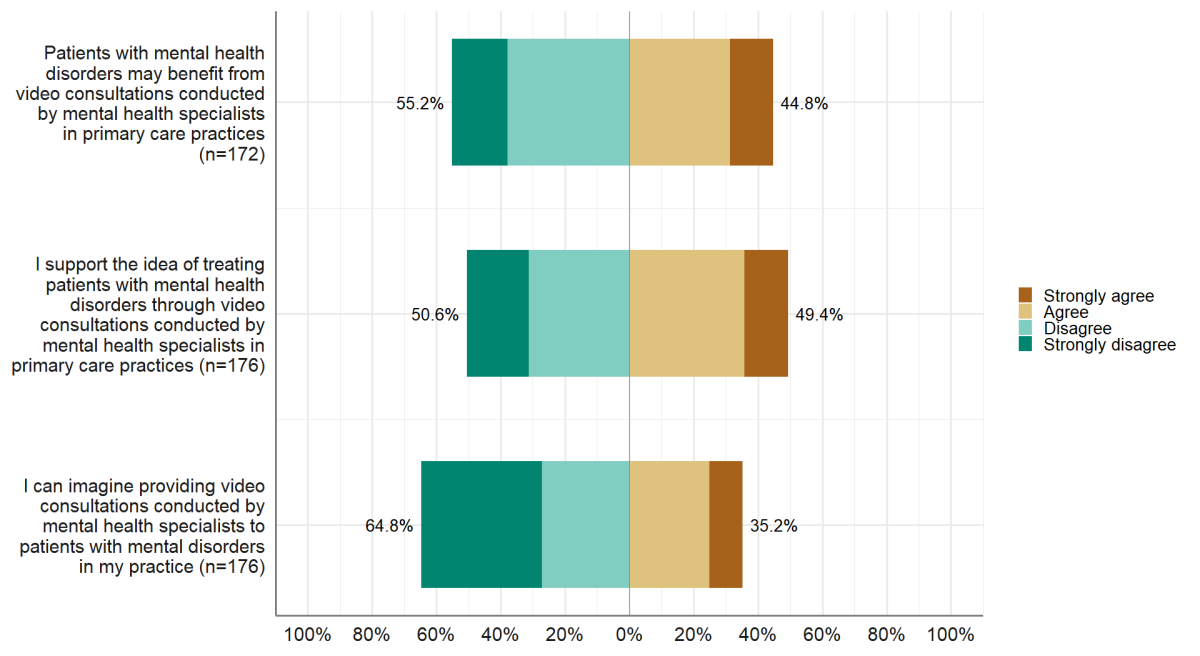
General practitioners’ agreement on benefit for patients, acceptance, and intent-to-adopt concerning mental health specialist video consultations.

We present the quantitative results followed by the qualitative characterization of the supporters and the nonsupporter of the MHSVC model. Finally, we interpret these findings with respect to their fit with the generalized categories of early adopters and nonadopters conceptualized in DOI (Figure 2).

### Main Results of the Quantitative Strand: Intent to Adopt Among GPs

#### Survey Participants

We invited 788 eligible GPs to participate in the initial survey (Multimedia Appendix 6 for the study flow chart). Eventually, 22.5% (177/788) GPs responded. Common reasons for nonparticipation were unknown (373/611, 61.3%), lack of interest (146/611, 23.6%), and time constraints (66/611, 10.8%). There were no statistically significant differences between nonresponders and responders concerning gender (*χ^2^*_1_=0.0; N=788; *P*=.96) and the degree of urbanization of the areas of practice were as follows: (*χ^2^*_2_=4.6; N=788; *P*=.10; Multimedia Appendix 7 for mosaic plots). There was no major difference in the average age (M=55.9 years, SD 8.8) of our sample compared with the average age of GPs at the country level (M=55.3 years), although no statistical comparison was possible owing to the missing SD for age at the country level. Table 2 shows the sociodemographic characteristics of the sample. After removing the 4 outliers, we included 173 cases in the multivariate analysis.

**Table 2:** Sample description for the quantitative strand.

Variable		Values
Female gender, n (%)		85 (48.0)
Age (years), mean (SD)		55.9 (8.8)
Additional qualification in addiction medicine and/or psychotherapy, n (%)^a^		19 (10.9)
Years in office-based practice, mean (SD)		18.2 (9.8)
**Type of practice, n (%)**		
	Solo practice	103 (58.5)
	Shared practice	63 (35.8)
	Group practice	10 (5.7)
**Number of physicians in the practice, mean (SD)**		
	Overall	2.1 (2.2)
	Full time	1.4 (0.9)
	Part time	0.7 (2.1)
**Degree of urbanization of the area the practice was located in, n (%)**		
	Cities (densely populated areas)	27 (15.3)
	Towns and suburbs (intermediate density areas)	111 (62.7)
	Rural areas (thinly populated areas)	39 (22.0)
**Average number of patients per quarter, n (%)**		
	<500	4 (2.3)
	501-1000	47 (27.3)
	1001-1500	54 (31.4)
	>1500	67 (38.9)
**Patients with mental health conditions per week, n (%)**		
	1-5	13 (7.4)
	5-10	32 (18.2)
	10-15	53 (30.1)
	>15	78 (44.3)
Designated room available for video consultations, n (%)		86 (49.1)

^a^Includes addiction medicine and/or psychotherapy. Multiple responses possible.

#### Anticipated Benefits for Patients, Acceptability, and Intent to Adopt

We assessed GPs’ attitudes concerning the anticipated benefit for patients from MHSVC, their acceptance of MHSVC, and their intent to adopt to MHSVC on ordinal agreement scales with 4 response categories, ranging from *strongly agree* to *strongly disagree* (Figure 2). Notably, while one in every 2 GPs stated having a designated room available for video consultations in their practice, only one in every 10 GPs indicated that they would be willing to offer video consultations.

#### Predictors for Intent to Adopt MHSVC (Proportional Odds Model)

To identify predictors for the intent to adopt MHSVC among GPs, we fitted a cumulative logit model for ordinal responses (Table 3). Applying the likelihood ratio test, we identified the availability of a designated room for video consultations in general practice as the only significant predictor. The deviance test statistic indicated that the model fitted adequately (χ^2^_467_=371.5; N=788; *P*=.99). The likelihood ratio test of the proportional odds assumption did not yield any evidence that this assumption was violated for any predictor variable.

**Table 3:** Proportional odds model for intent-to-adopt video consultations^a^.

Predictor variables		Coefficient^b^	SE^c^	z value	*P* value
Age of general practitioner		−0.014	0.017	0.830	.41
Additional qualification in addiction medicine and/or psychotherapy (ref: no)		−1.002	0.545	1.837	.07
**Type of practice (ref^d^** **: Solo practice)**					
	Shared practice	−0.386	0.389	0.990	.32
	Group practice	−0.545	0.738	0.738	.46
**Degree of urbanization of the area the practice was located in (ref: Cities [densely populated areas])**					
	Towns and suburbs (intermediate density areas)	−0.659	0.392	1.682	.09
	Rural areas (thinly populated areas)	−0.243	0.268	0.906	.37
**Average number of patients per quarter (ref: <500)**					
	<500	0.493	0.848	−0.581	.56
	501-1000	−0.115	0.625	0.185	.85
	1001-1500	−0.195	0.378	0.518	.61
Designated room available for video consultations (ref: no)		2.025	0.345	5.876	<.001

^a^Number of observations: 788. R²: 0.33 (Cox & Snell); 0.35 (Nagelkerke); and 0.15 (McFadden). Residual deviance: 371.52 on 467 degrees of freedom. Log-likelihood: 185.76 on 467 degrees of freedom. Akaike information criterion (AIC): 397.52. Bayesian information criterion (BIC): 437.49. Intercepts not displayed.

^b^Negative values indicate a lower likelihood of intent to adopt, positive values indicate a higher likelihood of intent to adopt.

^c^SE: standard error.

^d^ref: reference category.

### Main Results of the Qualitative Strand: Characterization of Supporters and Nonsupporters

#### Focus Group Participants

We identified 18 supporters and 56 nonsupporters among the 177 responding GPs. A total of 5 supporters (out of 10 who initially declared interest) and 1 nonsupporter (out of 4 who initially declared interest) eventually participated in the focus groups. Each of these 6 GPs joined a different focus group or participated in an individual interview and provided the data on which the following qualitative analysis was based (Table 4).

**Table 4:** Sample description for the qualitative strand.

Type of innovativeness	Gender	Age (years)	Additional qualification in addiction medicine and/or psychotherapy	Type of practice	Degree of urbanization of the area the practice was located in	Designated room available for video consultations	Focus group and interview
Supporter #1	Male	57	No	Solo	Towns and suburbs	Yes	# F1
Supporter #2	Male	60	Yes	Group	Rural area	Yes	# F3
Supporter #3	Male	53	No	Solo	Towns and suburbs	Yes	# F4
Supporter #4	Male	62	No	Solo	Towns and suburbs	Yes	# I2
Supporter #5	Female	60	No	Solo	Towns and suburbs	Yes	# I3
Nonsupporter	Female	56	Yes	Group	Towns and suburbs	No	# F2

In the following, we present shared characteristics among the 5 supporters and 1 nonsupporter. We then elaborate the specific characteristics of the supporters and the nonsupporter, highlighting major distinctions between the 2 groups.

#### Shared Characteristics of Supporters and Nonsupporters

Both, the 5 supporters and the 1 nonsupporter, strongly identified with the key role attributed to GPs in most health care systems and were highly committed to fostering the doctor-patient relationship unique to general practice:

The structure of a specialist’s practice is completely different from that of a GP. We are practically the last resort. We take everyone, unselected. This makes up the quality and uniqueness of our work.

Supporter #2

Who else in the medical field actually dares to proceed to this intimate level with patients? This is my advantage.

Supporter #1

Considering themselves as the principal health care providers for patients, GPs felt responsible for the early identification of mental health conditions. However, they perceived the referral of patients to specialist mental health care as very challenging. Specifically, they observed a high number of burdened patients faced long waiting times owing to the very limited availability of MHS:

There is a huge problem in routine care: When you have patients with an acute condition, referring them is always very difficult in my view.

Supporter #1

We still have the problem of getting patients referred. I have two psychotherapists, who I really like, but they work to capacity.

Nonsupporter

Continuously struggling with this ubiquitous supply-demand dilemma in their daily routine, all 6 GPs called for measures to increase the accessibility of specialist mental health services, for example, by scaling up cross-sectoral care models. Table 5 presents a joint display of the quantitative and qualitative results of the identified supporters and the nonsupporter.

**Table 5:** Joint display organized by the supporter and nonsupporter categories.

Group	Anticipated benefits for patients, mean (SD)	Acceptability^a^, mean (SD)	Intent-to-adopt criterion^a^	Participants’ statements (exemplary quotes from focus group or interview)
Supporter	1.28 (0.67)	1.28 (0.57)	1	Supporter #1: “Yes, if I have the possibility, to provide short-term video consultations for the patient at least for an initial therapy so that the patient does not have to wait endlessly until I can make an appointment with a suitable psychotherapist.” Supporter #3: “You have to see it like this: I would also benefit from it [the model], because it would help my patients.” Supporter #5: “I deal with hundreds of different diseases, which take up a lot of my time. But here I can get help that would also give me some relief.*”*
Nonsupporter	3.46 (0.57)	3.61 (0.49)	4	Nonsupporter: “It also depends a bit on the overall attitude. I am a more reserved type with these things, as you’ve already noticed. Others, who might start from the scratch, will be more interested.” Nonsupporter: “Let's put it this way: I think it's legitimate to try to use resources in a way that it is beneficial to most people. But I just don't think it's reasonable to shift patients in need from one provider to another.” Nonsupporter: “With patients being in an acute crisis, I am not sure if the video consultation works if they haven’t had experience with this setting before.”

^a^Lower values indicate higher anticipated benefits, higher acceptability, and higher intent to adopt, respectively.

#### Characteristics of Supporters

The supporters identified difficulties inherent in the organization of modern general. Specifically, they reported time constraints and a lack of qualification in mental health care. Supporters postulated that the MHSVC model would be effective at enabling low-threshold access to specialist care, primarily for patients presenting in general practice but also for themselves as GPs (eg, for brief case discussions):

I mean, we would lower the threshold significantly by offering the patient to only come to the familiar GP’s practice [to receive the video consultation] and nothing more. I think at some point during the treatment course, a moderate threshold is acceptable for the patient.

Supporter #3

Supporters were also open to new technology-based interventions and expected them to yield outcomes comparable with face-to-face treatments. They appreciated the possibility of immediately linking patients with MHS and, at the same time, saving the resources of the general practice:

I would be happy if I had such an instrument [the video consultations]. I could tell the patients who I consider to be in urgent need of treatment: ‘Listen, there's something, that you can do here for a few hours, at least temporarily. You may give it some thought’.

Supporter #4

If I know that I have someone in the background, I tell the patient, ‘Okay, I see your difficulties, but I can make some effort to get you a rapid appointment with a specialist’ knowing that the patient will be cared for.

Supporter #5

Supporters were genuinely interested in the practical implementation of the MHSVC, which reflected in detailed questions about which components the model would include and how it would be compatible with existing workflows in their practices:

My assistant could, so to speak, take the patient friendly by the hand and explain the technical details to him, where to press. They would also clarify whom the patient would turn to if there was a problem [during the video consultation].

Supporter #2

Although the supporting GPs demanded high usability from the video consultation platform and readily available technical support, they also took a solution-focused perspective on potential problems. Notably, the supporters expressed high confidence about being able to rapidly tackle unexpected difficulties during both the setup and the maintenance of the service:

As I said, so you just must seat the people in front of it [the screen] and see what happens. I would also be interested in experiencing that.

Supporter #4

Eventually, supporters reflected on the target population for video consultations and estimated the acceptability to be high with a few exceptions in certain patient groups. Specifically, supporters argued that older people would be less affinitive to the technology and struggle with it more often compared with younger patients:

Therefore, I think, the barrier to admitting that you have a psychological problem, this barrier is certainly very high for many. [...] This is certainly higher with the elderly than with younger ones.

Supporter #4

Overall, supporters (1) regarded video consultation as a mode of delivery equal to face-to-face settings, (2) anticipated specific advantages both for patients and themselves as GPs, and (3) tried to gain a comprehensive understanding of the practical ramifications of MHSVC.

#### Specific Characteristics of the Nonsupporter

The nonsupporter did not assume that video consultations could be effective for treating patients with mental health conditions. Specifically, she argued that not meeting in-person would entail the risk of specialists missing nonverbal cues and preclude physical contact, for example, through common gestures, the recognition of which, in her opinion, was essential for health care to be effective:

There are so many small things that you can notice, and they would, of course, be missed during the video consultation.

Nonsupporter

The nonsupporter advocated firmly that a trusting therapeutic relationship could only be developed in the traditional face-to-face setting and saw no room for new, technology-facilitated service delivery models. Arguing from a problem-oriented perspective, the nonsupporter displayed a fundamental disapproval for video consultations:

As I said, I think that it might work for some, but generally it is very different from sitting across from someone. Then, you get information that you do not get over the screen.

Nonsupporter

At the health care system level, the nonsupporter considered the MHSVC model to be ineffective in increasing access to specialist mental health care. Specifically, she expected a shift of MHS/personnel resources away from specialist in-person care to virtual care models. From her perspective, there would be less workforce available in specialist mental health care than today:

I am going to be very heretical now: If the need was better met, this project would not even have come up, would it?

Nonsupporter

At some point, the nonsupporter referred to her self-concept, characterizing herself as being reluctant to support and adopt new health care technologies. She clearly wanted to preserve her reserved stance toward change:

I am a rather more reserved type with these things. [...] I personally feel that I would really like to keep myself as I am. And maybe I do not have this readiness for change in me; I am rather reserved.

Nonsupporter

Like the supporters, the nonsupporter emphasized that older people would be less familiar with the technology, and therefore, inevitably display a negative attitude toward video consultations:

I imagined my mother sitting there at the age of 87. [...] And I cannot imagine that the elderly really feel comfortable in this setting, but rather the younger and middle-aged perhaps.

Nonsupporter

Beyond that, the nonsupporter expected that the acceptability of MHSVC in patients would *generally* be rather low. However, she also expected a small proportion of younger patients with mild disorders to be likely to benefit from the model. Overall, the nonsupporter regarded integrated video-based mental health care as ineffective because she expected (1) a rather low acceptability by patients and (2) that MHSVC would be ineffective owing to the lack of nonverbal cues and face-to-face interaction, the latter being essential for mental health care in her consideration.

### Integration of Results and Comparison With Rogers’ Diffusion of Innovations Theory

We found some evidence supporting the generalized adopter categories in the DOI. Supporters in our sample showed both a general openness toward technology and a great ability to deal with uncertainty related to the relatively new concept of MHSVC. Specifically, supporters tried to develop forward-looking strategies for potential problems (eg, technical failures) potentially impeding working routines in general practice. Such a solution-focused stance is typical of early adopters, as conceptualized in the DOI. Moreover, supporters in our study were less dogmatic and expressed a more favorable attitude toward change compared with the nonsupporter, an observation that is also in line with the generalized DOI categories of early adopters and nonadopters, respectively. In contrast, the nonsupporter in our sample anticipated several problems (eg, the elderly being less open-minded) but was not concerned with potential solutions. Rather, she explicitly highlighted her preference for preserving the status quo and revealed an attitude based on values tied to the established in-person standard. Although supporters were very interested in the success of the MHSVC model as they expected future benefits for patients and themselves, the nonsupporter’s point of reference was the past (what has been done ever since). When exploring links between innovativeness and sociodemographic characteristics, we found no support for the hypothesis of the DOI that early adopters have larger units (absorbing the loss from occasional innovation failures) compared with late adopters. Rather, the nonsupporter in our sample ran a large practice (>1500 patients on average per quarter). However, in line with the DOI, we found no differences in age between the various categories of adopters. We did not explicitly address other characteristics related to innovativeness according to the DOI and could therefore not evaluate evidence for early adopters being opinion leaders, adopting new ideals as a result of information exchange with interpersonal networks or exhibiting greater empathy.

## Discussion

### Principal Findings

This study found that (1) about one in every 2 GPs assumed that patients would benefit from the MHSVC service model, (2) about one in every 3 GPs intended to adopt such a model, (3) the availability of a designated room was the only significant predictor of intent to adopt in GPs, and (4) supporting GPs also expected to save time in their practice and took a solution-focused perspective on the practical implementation of MHSVC. Furthermore, the GP who did not support the MHSVC model assumed that no effective therapeutic relationship could be established with patients using video consultations. Finally, we found preliminary evidence that the characteristics of supporting and nonsupporting GPs in the context of MHSVC corresponded well with the generalized adopter categories conceptualized in the DOI.

### Limitations

Our findings must be interpreted considering some shortcomings. First, concerning the quantitative results, this was a cross-sectional study that did not allow inferring any temporal or even causal associations between attitudes toward video consultations and actual behavior. To illuminate the direction of the observed associations, longitudinal studies (randomized controlled trials or prospective cohort studies) are needed. Second, nonresponse bias undermining the generalizability of the findings is a ubiquitous challenge, particularly in general practice research [47]. Nevertheless, our response rate was somewhat higher than the usual 20% expected in postal questionnaires [48]. Although we were only able to include 2 variables in the nonresponder analysis, we did not find any statistically significant differences between nonresponders and responders. Moreover, the mean age of our sample was comparable with the mean age of GPs in Germany. These findings indicate that the sampling error was rather low and that we obtained a composite profile of the larger population.

With respect to the qualitative findings and given the limited number of 6 individuals, our findings are preliminary. However, recruitment of nonadopters for studies of interventions that do not support is usually particularly challenging. At any rate, the integration of quantitative and qualitative data in our study contributes to the credibility of our findings. As is characteristic of preimplementation studies, none of the participants had practically conducted MHSVC before participating in the focus group. Instead, our study collected pretrial observations focusing on the behavior of intended users and their perspectives. Therefore, some GPs may revise their attitude toward the intervention model after the actual implementation. However, by describing the model in detail accompanied by a video clip, we encouraged the participants to gain a comprehensive understanding of the model. Finally, the classification of adopters is a simplification that inevitably neglects information on individuals. In the true sense, innovativeness is a continuous variable with no sharp cut points. Moreover, recent work has called some generalizations of the DOI into question, elucidating that nonadopters often very consciously refute evidence-based practices that they do not find to be relevant for their everyday psychosocial practice [49]. Nevertheless, generalizations proposed in the DOI are of tremendous heuristic value for understanding human behavior change and tailoring audience segmentation strategies [24,50]. Future studies should investigate larger sample sizes and collect performance data on overt behavioral changes, which may reveal additional characteristics of supporting and nonsupporting GPs in the context of MHSVC.

### Comparison With Previous Work

From a macro-level perspective, the frequency of adoption of consequential innovations begins slowly before accelerating through spread in the professional community, following an S-shaped pattern for the cumulative number of adopters over time [24,51,52]. Indeed, considering our response rate and the frequency of respondents who indicated that they would adopt MHSVC, the rate of adoption in our sample amounts to 7.9%, which is similar to previous findings [53]. Notwithstanding, the proportion of GPs intending to adopt MHSVC in their own practice corresponds well with increasing utilization rates of telehealth in primary care and specialist health care services (eg, mental health facilities and community-based federally qualified health centers in the United States) [19,54-56]. At a microlevel, the importance of preimplementation assessments of barriers to change, as anticipated by clinicians, has been emphasized frequently [57]. Specifically, by assessing the current provider environment and characterizing early adopters based on the generalizations of the DOI, our study will facilitate the selection of GPs for feasibility and effectiveness trials evaluating MHSVC as a cornerstone of primary care mental health [58-60]. Following an audience segmentation strategy [50], the early and late majority should be targeted only in the next step. In this regard, the characteristics of GPs exhibiting high innovativeness or a high tendency to adopt MHSVC, correspond well in our study not only with the generalized type of the DOI [24] but also with the description of early adopting GPs in other areas of primary care mental health [33]: GPs in our study regarded MHSVC as a sound opportunity for addressing common mental health care problems (eg, by increasing treatment initiation and engagement) and expected the MHSVC to fit logistically with the workflows in their practices and, in some instances, even produce some workload relief. This finding is somewhat in contrast to observations from a study on web-based consultations to foster communication between GPs and specialists for seamless care coordination [29]. These consultations, similar to web-based consultations provided by GPs themselves [31], not only proved to be difficult to integrate into existing workflows but also lacked reimbursement strategies. However, in our study, MHSVC was conceptualized to take place between patients and MHS and to focus on the communication between MHS and GPs using written short reports. This format may be more feasible in a busy general practice environment where reimbursement opportunities for collaborative work are still limited and less disruptive digital services are welcomed more readily [32]. In accordance with Rogers’ DOI, the early adopters in our study appeared to be somewhat less dogmatic with respect to in-person visits and have a more favorable attitude toward change compared with late adopters [24]. Our finding that GPs not supporting MHSVC in general practice place much value on face-to-face encounters and entertain concerns about loss of nonverbal and social presence cues has been reported previously [61-64]. Indeed, a strong preference for in-person communication is the main reason why clinicians do not use mental health services via videoconferencing [65,66]. In contrast, current evidence demonstrates that patients using clinical videoconferencing visits are comfortable and satisfied with this mode of care delivery [67]. They experience the sessions to be as beneficial as in-person visits [68,69]. Moreover, there is some evidence that clinicians have more concerns about alliance than patients do [70]. Concerning socioeconomic characteristics, early adopters in our study, in contrast to the DOI hypothesis, did not maintain larger units compared with late adopters. As GPs in our study were only questioned on attitudinal change, it seems plausible that early adopters did not account for the potential need to absorb financial losses in case MHSVC could not be implemented successfully. However, prices for videoconferencing systems have decreased significantly in recent years.

### Conclusions

GPs’ readiness for implementing the anticipated MHSVC delivery model in general practice is considerable, as this model may be suitable for addressing the most pressing needs of both patients and clinicians. Currently, there is a significant proportion of GPs who may function as (1) early adopters with solid buy-in in future feasibility and effectiveness trials and (2) key stakeholders facilitating the spread of MHSVC through information exchange in interpersonal networks. Indeed, we have just completed a feasibility trial (Trial registration: DRKS00015812), which has yielded promising results and initiated a full-scale trial (NCT04316572). Beyond that, future work should focus on educational measures to facilitate the implementation of the model for the large number of GPs who are hesitant to this day (early and late majority). Interventions targeting acceptance and implementation should account for the clinicians’ competencies (eg, technology commitment) and information needs (eg, how MHSVC works and how to manage emergencies, benefits, and limitations) to increase their comfort with videoconferencing as a treatment modality [71]. Although the number of patients in need in remote and rural areas may be high [54,72], the demand for MHSVC is dependent on the willingness of GPs caring for those patients to refer them.

## Appendix

Multimedia Appendix 1 Search strings for the systematic review.

Multimedia Appendix 2 Consolidated Criteria for Reporting Qualitative Research checklist.

Multimedia Appendix 3 Questionnaire for general practitioners.

Multimedia Appendix 4 Semistructured question guide for focus groups and telephone interviews.

Multimedia Appendix 5 Description of the coding system.

Multimedia Appendix 6 Study flowchart.

Multimedia Appendix 7 Mosaic plots for the nonresponder analysis.

## Abbreviations

DOI: Diffusion of Innovations Theory
GP: general practitioner
MHS: mental health specialists
MHSVC: mental health specialist video consultations
PROVIDE: ImPROving cross-sectoral collaboration between primary and psychosocial care: An implementation study on VIDEo consultations
