# Supplementary material for: Intent to Adopt Video-Based Integrated Mental Health Care and the Characteristics of its Supporters: Mixed Methods Study Among General Practitioners Applying Diffusion of Innovations Theory
Source: JMIR Ment Health. 2020 Oct 15;7(10):e23660. doi: 10.2196/23660 (PMC7654505; doi:10.2196/23660)
Supplement: Multimedia Appendix 1 [file mental_v7i10e23660_app1.docx]

**APPENDIX 1. SEARCH STRINGS FOR THE SYSTEMATIC REVIEW**

**MEDLINE:**

**Filters:** Fields: Title/Abstract; Species: Humans; Languages: English, German

**Search string (including filters):**

"early adoption"[TIAB] OR "early adopter"[TIAB] OR "early adopters"[TIAB] OR "early adopting"[TIAB] OR "diffusion of innovation"[TIAB] AND ("humans"[MeSH Terms] AND (English[lang] OR German[lang]))

**Link:** <https://www.ncbi.nlm.nih.gov/pubmed/?term=%22early+adoption%22%5BTIAB%5D+OR+%22early+adopter%22%5BTIAB%5D+OR+%22early+adopters%22%5BTIAB%5D+OR+%22early+adopting%22%5BTIAB%5D+OR+%22diffusion+of+innovation%22%5BTIAB%5D+AND+(%22humans%22%5BMeSH+Terms%5D+AND+(English%5Blang%5D+OR+German%5Blang%5D))>

**No. of records:** 1112 (as of August 19^th^, 2020)

**WEB OF SCIENCE:**

**Filters:** Fields: Topic; Languages: English; Document Type: Article

**Search string (including filters):**

(((TS="early adoption") OR (TS="early adopter") OR (TS="early adopters") OR (TS="early adopting") OR (TS="diffusion of innovation") )) *AND* LANGUAGE: (English) *AND* DOCUMENT TYPES: (Article)

**Link:** n/a

**No. of records:** 2.832 (as of August 19^th^, 2020)
