# Supplementary material for: Intent to Adopt Video-Based Integrated Mental Health Care and the Characteristics of its Supporters: Mixed Methods Study Among General Practitioners Applying Diffusion of Innovations Theory
Source: JMIR Ment Health. 2020 Oct 15;7(10):e23660. doi: 10.2196/23660 (PMC7654505; doi:10.2196/23660)
Supplement: Multimedia Appendix 3 [file mental_v7i10e23660_app3.docx]

**APPENDIX 3. QUESTIONNAIRE FOR GENERAL PRACTITIONERS**

**Personal details of the general practitioner**

| **1. Sex:** | Male Female |  |
| --- | --- | --- |

**2. Year of birth (e.g., 1970):**

**3. Since when have you been a practice-based general practitioner? (e.g., since 1992):**

|  | Not applicable |  |
| --- | --- | --- |

**4. If applicable, which of the following** **additional mental healthcare qualifications do you hold? (please mark all options from the list that apply)**

| Psychotherapy | Psychosomatic basic care | Addiction Medicine |
| --- | --- | --- |

**Characteristics of the general practice**

**5. What type of practice are you running/working with?**

| Single practice | Group practice | Shared practice |  |
| --- | --- | --- | --- |
| **6. How many family physicians work in your practice?** | | | |

| Part-time | Full-time |  |
| --- | --- | --- |

| **7. What is the average number of cases per quarter?** | | | |
| --- | --- | --- | --- |
|  | | | |
| < 500 | 501 – 1000 | 1001 – 1500 | > 1500 |

| **8. On average, how many patients with mental health disorders do you see per week?** | | | |
| --- | --- | --- | --- |
|  | | | |
| 1-5 | 5-10 | 10-15 | > 10-15 |

**Intent to adopt mental health video consultations in the practice**

| **9. Do you think that patients with mental health disorders may benefit from the implementation of video consultations conducted by mental health specialists in primary care practices?** | | | |
| --- | --- | --- | --- |
|  | | | |
| Strongly agree | Agree | Disagree | Strongly disagree |

| **10. Would you support the idea of treating patients with mental health disorders through video consultations conducted by mental health specialists in primary care practices?** | | | |
| --- | --- | --- | --- |
|  | | | |
| Strongly agree | Agree | Disagree | Strongly disagree |

| **11. In principle, can you personally imagine providing video consultations conducted by mental health specialists to patients with mental disorders in your practice?** | | | |
| --- | --- | --- | --- |
|  | | | |
| Strongly agree | Agree | Disagree | Strongly disagree |

| **12. Would it be possible to conduct video consultations in a confidential treatment setting in your practice?** | | |
| --- | --- | --- |
|  | | |
| Yes | No |  |

**4. Interest in participating in a focus group**

**Yes, I am interested in participating in a focus group discussing video consultations provided by mental health specialists in primary care practices: __________________________ (e-mail or phone no.)**
