# Supplementary material for: Intent to Adopt Video-Based Integrated Mental Health Care and the Characteristics of its Supporters: Mixed Methods Study Among General Practitioners Applying Diffusion of Innovations Theory
Source: JMIR Ment Health. 2020 Oct 15;7(10):e23660. doi: 10.2196/23660 (PMC7654505; doi:10.2196/23660)
Supplement: Multimedia Appendix 4 [file mental_v7i10e23660_app4.docx]

**APPENDIX 4. SEMISTRUCTURED QUESTION GUIDE FOR FOCUS GROUPS AND TELEPHONE INTERVIEWS** *(Finalized version as of August 2017)*

**A. Current health care of patients with mental health conditions in primary care**

- Considering outpatient services, what do you think of the current health care of patients with mental health conditions?
  - To what extent do you perceive gaps in the current health care system?
  - If applicable, how do you personally tackle with these gaps?
- How would you describe the typical patient suffering from a mental health disorder in your practice?
  - If applicable, how would you describe any peculiarities entailed in health care delivery for patients with mental health conditions?
- How would characterize sound health care delivery for patients with mental health conditions?
- If applicable, do you benefit from a local network involving mental health specialists in outpatient care?

**B. Introduction of the PROVIDE intervention model, implementation potential und compatibility (e.g., readiness for change)**

- What do you think of the proposed treatment model in the PROVIDE project?
- To what extent do you expect to benefit from such a model in your personal working environment?
- Which patient groups seem suitable for receiving treatment under the proposed model?
  - What could be reasons that patients might not make use of the proposed video consultations?
- From your point of view, are family physicians currently willing to employ the proposed treatment model?

**C. Possible factors that promote or inhibit implementation of video consultations in primary care**

**(Determinants of practice)**

- What are the challenges for implementing video consultations in primary care? For
  - patients
  - health care personnel
    - in terms of time, location, organization and training
- What are the benefits of implementing video consultations in primary care? For
  - patients
    - To what extent do you expect patients with mental health conditions to benefit from the treatment model?
  - health care personnel
    - To what extent do you expect to benefit from the treatment model? (e.g., regarding workload)
- How would you define the within-practice allocation of roles between nurses, family physician and mental health specialist?
- Is it possible to conduct video consultations in a confidential treatment setting in your practice? If applicable, how can a confidential treatment setting be organized?
- How could the video consultations be scheduled in your practice?

**D. Specific parameters and necessary adaptions of the PROVIDE treatment model**

- Which models of financial remuneration do you consider appropriate?
- If applicable, what would you suggest to further optimize the treatment model

**E. Interview termination**

- Do you have any further questions?
- Are there any remaining aspects important to you that we have not addressed at this point?
