# Supplementary material for: Intent to Adopt Video-Based Integrated Mental Health Care and the Characteristics of its Supporters: Mixed Methods Study Among General Practitioners Applying Diffusion of Innovations Theory
Source: JMIR Ment Health. 2020 Oct 15;7(10):e23660. doi: 10.2196/23660 (PMC7654505; doi:10.2196/23660)
Supplement: Multimedia Appendix 5 [file mental_v7i10e23660_app5.docx]

**APPENDIX 5. DESCRIPTION OF THE CODING SYSTEM**

| ***Key domain*** | ***Definition*** | ***Subdomains*** | ***Supporting quotes*** |
| --- | --- | --- | --- |
| *Perceptions of mental health care* | *Factors regarding the sufficiency of mental health care supply* | *Problems in mental health care* | *Supporter #4: “In general, it is an absolute disaster. We have great difficulties when trying to refer patients to psychotherapists and when you're very lucky, after six months, seven months, eight months you get an appointment.”* |
|  |  | *Diagnosing and therapy of psychological problems in primary care (e.g. barriers of uptake of therapy)* | *Supporter #3: “It is also the case that patients often refuse mental health treatment. They often don't want to see a psychotherapist in the first place. They are afraid of it.”*  *Supporter #3: “After all, many women or older women still do not have a driving license or have not driven a car for 35 years. If they don't get something [a placement with a mental health specialist] in the city, it surely gets very difficult for them.”* |
|  |  | *Typical patient suffering from a mental health disorder in your practice* | *Supporter #2: “There are often young people who can’t find a foothold in life and do not attend school or vocational training. They often get a severe depression related to this instability. Often also comorbid with drug consumption.”* |
| *GP’s role in health care* | *Factors describing the GP’s importance in medical care* | *GP’s key role (e.g. communication with the patient and high engagement)* | *Supporter #2: ”The unique thing about us GPs is that we do not see new people every day, but to a large extent the same people who come back to us every few weeks. (...)it's about accompanying these people.”* |
| ***Key domains*** | ***Definition*** | ***Subdomains*** | ***Supporting quotes*** |
| *Practicability and Integration in the daily workflow* | *Description of the feasibility and advantages of the integration of video consultations in daily practice* | *Role of the GP in the project (e.g. relationship GP-therapist, differences between GP and therapist)* | *Supporter #1: “I think we would at least have to be nearby and available during the time [of the video consultation.”*  *Supporter #3: “it would be great if we could just get a short feedback. Better a rapid short one than a referral letter after two months.”* |
|  |  | *Outcome expectancy (e.g. patient doesn’t receive good therapy, patient receives sound treatment, possibility of relief for GP)* | *Supporter #3: “I would like to offer something to my patient. [With the video consultation] I could really offer something, that could make them feel better. And that’s why I treat patients.”* |
| ***Key domains*** | ***Definition*** | ***Subdomains*** | ***Supporting quotes*** |
| *Openness toward new innovations* | *To what extent are the GPs open for new innovations* | *Forward thinking* | *Supporter #3: “Or a traffic light system, red, green, yellow, along the lines, everything is going well [feedback of therapist] or, watch out, terrible. Be careful, you have to be active now. This would make sense.”* |
|  |  | *Interest for the video consultations (e.g. mode of delivery doesn’t matter)* | *Supporter #2: “Uh, that he [the patient] gets offered good care, care that makes sense, that picks him up where he stands, that enables him become more active. So that he can see that the change is it worth. […] That's the special feature of this model – that's how I see it.”* |
|  |  | *No readiness for change (e.g. mode of delivery does matter, no interest in change)* | *Non-supporter: “Although, as I said, I think that might work for some people, but basically, it's different when you sit opposite someone. Because you get information that you don't have across/through the screen.”* |
| *Others* |  | *Acceptance in the target group* | *Supporter #5: “Young people will be open to it, but the older ones, they will have problems, of course. I suppose so.”* |
