# Supplementary figures and images for: Intent to Adopt Video-Based Integrated Mental Health Care and the Characteristics of its Supporters: Mixed Methods Study Among General Practitioners Applying Diffusion of Innovations Theory
Source: JMIR Ment Health. 2020 Oct 15;7(10):e23660. doi: 10.2196/23660 (PMC7654505; doi:10.2196/23660)

**APPENDIX 6. STUDY FLOW CHART**


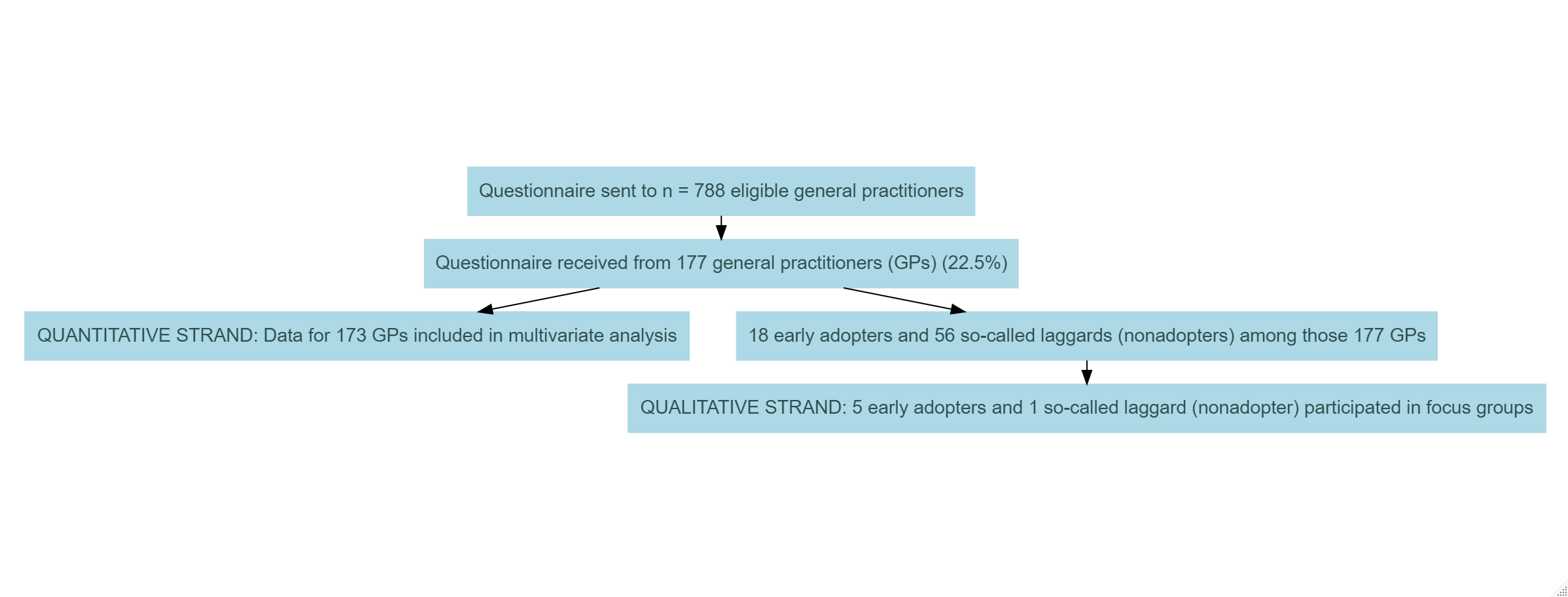

Supplement: Multimedia Appendix 6 [file mental_v7i10e23660_app6.docx]
