# Supplementary material for: Intent to Adopt Video-Based Integrated Mental Health Care and the Characteristics of its Supporters: Mixed Methods Study Among General Practitioners Applying Diffusion of Innovations Theory
Source: JMIR Ment Health. 2020 Oct 15;7(10):e23660. doi: 10.2196/23660 (PMC7654505; doi:10.2196/23660)
Supplement: Multimedia Appendix 7 [file mental_v7i10e23660_app7.docx]

**APPENDIX 7. MOSAIC PLOTS FOR THE NONRESPONDER ANALYSIS**

*
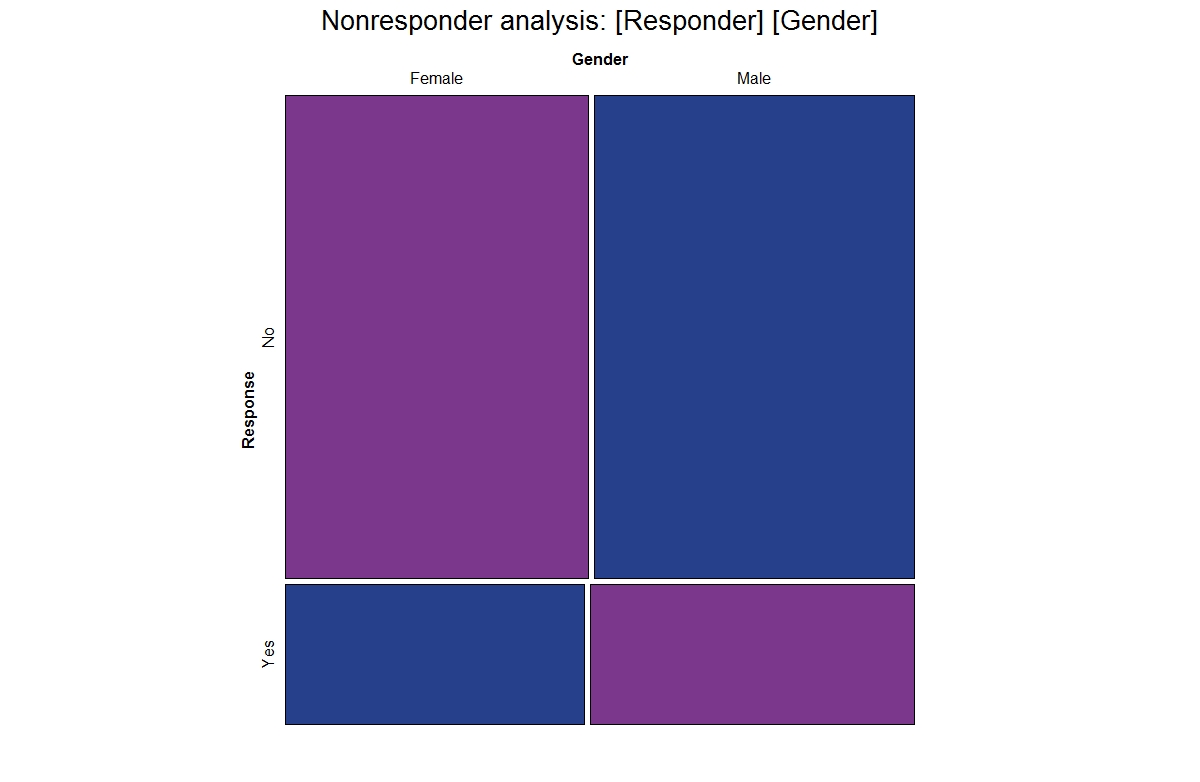
*

*Figure A1. Mosaic plot for the nonresponder analysis concerning gender*

*
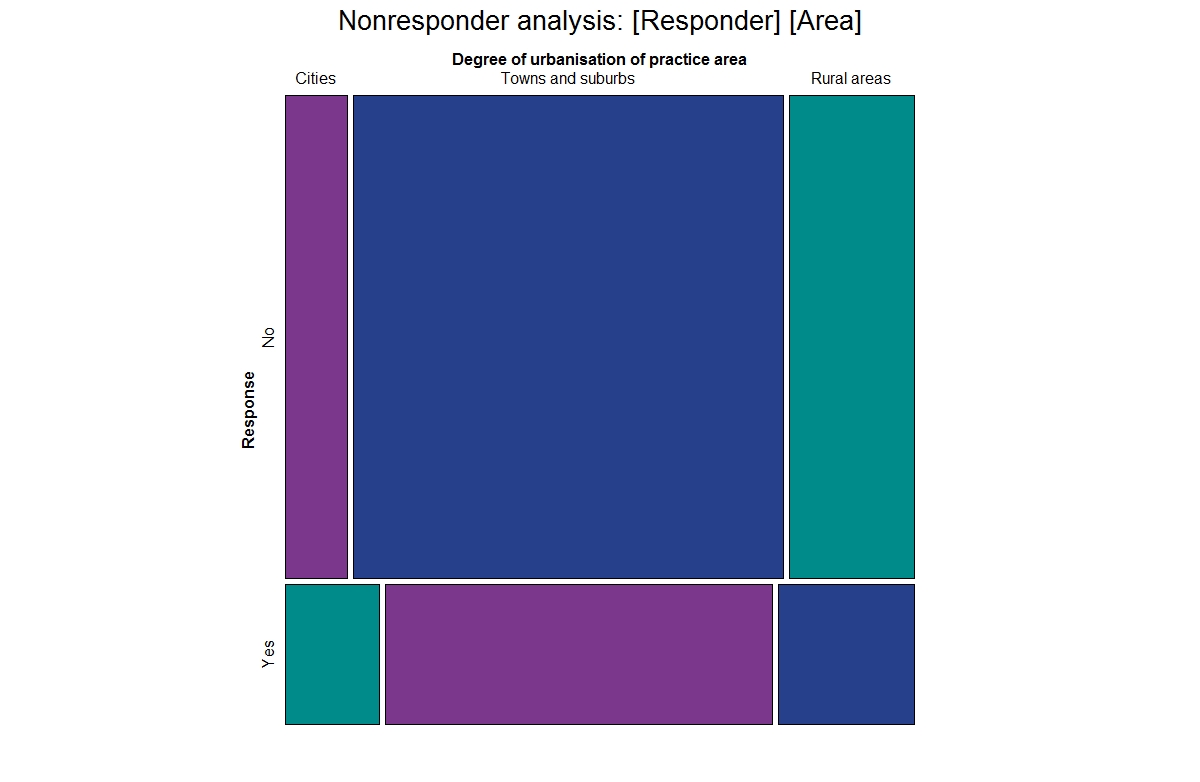
*

*Figure A2. Mosaic plot for the nonresponder analysis concerning gender*
